# An innovative infection method for the accumulation of viral nanoparticles in *Nicotiana benthamiana*

**DOI:** 10.3389/fpls.2025.1727190

**Published:** 2025-11-26

**Authors:** Kristina Ljumović, Anthony Rosa, Alessia Raneri, Matteo Ballottari, Linda Avesani, Nico Betterle

**Affiliations:** 1 Department of Biotechnology, University of Verona, Verona, Italy; 2 Diamante SB srl, Verona, Italy

**Keywords:** *Nicotiana benthamiana*, plant molecular farming, TBSV, viral nano particles, transient expression, hydroponic, spraying infection

## Abstract

Tomato Bushy Stunt Virus (TBSV) naturally infects tomato plants, although it can also infect other plant species, such as *Nicotiana benthamiana*, a known model system in plant molecular farming. In the presented work, a novel system for TBSV infection of *Nicotiana benthamiana* plants, designed to produce nanomaterials, was developed and optimized based on a simple foliar spray, without the use of surfactants. Up to now, the standard procedures for the viral infection have been syringe or vacuum infiltration, which are a time-consuming manual procedure or requiring expensive machinery, respectively. The spraying method was chosen because it could be implemented in industrial conditions, such as vertical farms, where spraying systems are already present or can be easily installed at a low cost. In this work, as a proof of concept, a wild type and a modified version of TBSV construct, which generated a viral nanoparticle (VNP) exposing a small 12 aa-domain Liprin alpha 1 protein (Lip1) on each capsid protein, were successfully expressed in *Nicotiana benthamiana* plants. Specifically, VNP displaying Lip1 is a candidate for the treatment of rheumatoid arthritis. After 7 days of incubation, signs of viral infection were visible in the infected plants, while prolonged incubation time to 8 days significantly increased the accumulation of VNPs. The infection method described here offers straightforwardness and scalability of plant molecular farming, representing an efficient solution for the complexity of the conventional infection process.

## 1 Introduction

Viruses are infectious agents programmed to deliver their nucleic acids into the hosts, such as plants, humans, and animals, enabling their replication and production of new viral copies. This strategy has several properties that can be further applied in various fields, including biotechnology, agriculture, medicine, and nanotechnology (Wen and Steinmetz, 2016; Nkanga and Steinmetz, 2021). Auto-assembly of one or more protein subunits forms viral capsids, establishing VNPs that are homogenous in shape and dimension. On the capsid subunits, it is possible to attach peptides of interest both via genetic engineering and via chemical reactions (Sánchez et al., 2013). VNPs can be based on bacteriophages, mammalians, and plant viruses. In particular, plant VNPs have all the characteristics mentioned above and are not pathogenic to mammals such as humans.

The production of plant VNPs can be achieved by molecular farming, which consists in the production of recombinant proteins or secondary metabolites of interest using plants as biofactories. To date, plant VNPs have been developed for the production of therapeutic agents and immunostimulants (Rybicki, 2020), as well as for applications in bioimaging and cancer therapies (Zhou et al., 2023). Specifically in the case of immunostimulants, applications of recombinant plant VNPs consisted in the surface exposure of immunodominant peptides associated with autoimmune diseases.

Rheumatoid arthritis is a systemic disease that comprises progressive synovial inflammation (Majithia and Geraci, 2007). Recently, it has been shown that Lip1 peptide is immunodominant in patients with Rheumatoid Arthritis (Bason et al., 2021). Such a peptide, composed of 12 amino acids, was successfully expressed on the surface of TBSV Tomato Bushy Stunt Virus (TBSV) capsid (Zampieri et al., 2020). Specifically, Lip1 was fused to the viral coat proteins, yielding TBSV.pLip. TBSV usually infects tomato plants, but successful infection of *Nicotiana benthamiana* plants has also been reported (Zampieri et al., 2020).

Tomato bushy stunt virus (TBSV), a member of the Tombusvirus genus, is a non-enveloped plant virus approximately 30 nm in diameter, composed of 180 identical copies of its coat protein (CP). While wild-type TBSV is not known to infect humans or other mammals, it is pathogenic to a range of plant species, including tomato and various horticultural crops. The virus has a broad geographic distribution, having been reported in Western and Central Europe, North Africa, Argentina, Canada, and Mexico (Tomlinson et al., 1982).

TBSV transmission occurs primarily through mechanical means, including direct inoculation and grafting. It can also be transmitted via seed at low frequencies and potentially through pollen-mediated pathways, although it is not spread by simple plant-to-plant contact. Notably, TBSV can persist in soil environments, from which it may be taken up by susceptible host plants (Martelli et al., 1988). Its use for biotechnological applications is conducted under strict biological containment, by maintaining and propagating the virus in laboratory Escherichia coli strains that are non-pathogenic to humans and plants. Biological containment also involves limiting environmental transmission by treating soil material as infectious waste. An additional strategy to prevent horizontal transmission is to inhibit flowering in infected plants, thereby reducing the risk of pollen- and seed-mediated spread.

The ability of TBSV to infect *N. benthamiana* is due to the presence of CP and p19 protein. The CP protein can be modified to attach a peptide of interest to its C-terminus (Marchetti et al., 2023). The presence of p19 protein is required to suppress virus-induced gene silencing in plants, allowing the virus to systemically infect the host (Qu and Morris, 2002).

Conventional techniques for the transient expression of heterologous proteins in plants rely on the use of *Agrobacterium tumefaciens* and manual or automated infiltration methods (Zampieri et al., 2020). Manual methods include syringe infiltration of individual leaves; this methodology is time- and labor-consuming, and it is also prone to technical errors. Conversely, automated methods use expensive machinery to simultaneously infect a large number of plants (eg. vacuum pumps for infiltration). These processes have a significant impact on the capital investment and operational costs of the molecular farming facility (Huebbers and Buyel, 2021). Moreover, an experienced workforce is needed for these operations, which can further increase operational costs. Considering these challenges, there was a need for the development of innovative and less expensive infection methods.

In this work, a novel approach to produce VNPs of interest in *Nicotiana benthamiana* is presented. Such an approach relies on spraying viral sap on top of the plant biomass, simplifying the process of plant infection with the TBSV virus. The latter was either wild-type or TBSV.pLip. Several tests were conducted to optimize the accumulation of viral coat proteins in plant biomass. In conclusion, this work describes an alternative to time-consuming manual infiltration methods, which are currently considered as gold standard in plant molecular farming. Furthermore, the spraying infection method offers the opportunity for automation of the production system, leading to a potential decrease in operational costs.

## 2 Materials and methods

### 2.1 Plant material and growth conditions

*N. benthamiana* plants were grown hydroponically using the formulation Idrofill Base (https://k-adriatica.it/eng/products/hydroponics/idrofill-base; K-Adriatica, Italy), ~2 g/L, supplemented with 50 mg/L Sequifill 6.0T SS, a Fe-EDDHA-based product, as an iron source (https://k-adriatica.it/eng/products/meso-and-microelements/sequifill-6.0t-ss; K-Adriatica, Italy). The electrical conductivity (EC) of the hydroponic solution (HS) was set at 2.4 mS/cm and the pH was adjusted at ~5.5. These two parameters were measured using a DiST4 EC tester and a Checker pHmeter (Hanna Instrument, USA). The nutrient solution of the plants kept in the growth chamber was refilled every week, and mild air bubbling was supplied to ensure oxygenation of the HS.

The seeds of *N. benthamiana* plants were put on plastic plates with wet paper towels for germination. Such plates were kept for 4 days at 25 °C in dark conditions. After germination, seedlings were placed in plastic plugs containing inert jute (Holland BioProducts, The Netherlands) as support for plant rooting. Such plugs were then put in specific metal grids and trays for hydroponic cultivation (Ono Exponential Farming, Italy) (**Supplementary Figure S1**). The growth trays containing the seedlings were placed under light at ~200 µmol*m^-2^s^-1^, with a humidity of ~60% and a temperature of ~23 °C. The jute supports were moisturized daily with a nutrient solution to prevent them from drying out. 400 plants/m² were cultivated, and the hydroponic solution was added on demand to prevent plant dehydration during lab-scale hydroponic cultivation in a controlled chamber. The volume of nutrient solution used in the trays was ~4 L. Plants were cultivated for an average of 30 days.

### 2.2 Infection methods

A fine mist sprayer (**Supplementary Figure S2**) was used to infect the plants, and two middle leaves of a ~25-day-old plant (leaf length 5–6 cm) were sprayed with ~400 µL of viral sap or purified particles each. Viral sap consists of sap from homogenized TBSV-infected plants containing VNPs (Esteves et al., 2021). Viral sap was obtained by homogenizing plant biomass in a buffer solution. Such biomass was prepared starting from previously infected plant material, which was stored at -80 °C. The homogenization occurred in 50 mM PBS buffer pH 7.4, using an ice-cold mortar, with a 1:5 or 1:10 ratio (g/mL) of plant biomass to buffer solution. The supernatant was collected after centrifugation and used for the infection.

In the case of the positive control samples, the leaf surface was covered with celite (Merck Life Science, Italy), causing first mechanical damage, and then 40 µl of viral sap was spread over the leaf. After the infection, plants were immediately placed in an incubation chamber for the required incubation time. Plant material was collected after 7-, 8-, 9-, and 10-days post-infection (dpi).

In all the infection tests, second-generation leaf material provided by Diamante SB srl (Italy) was used as a starting viral sap. Specifically, first-generation leaf material was obtained by introducing the DNA construct harboring viral genes into the plant through agroinfiltration. Consequently, second-generation plants were infected with viral sap extracted from the leaves of first-generation plants.

### 2.3 Extraction and purification of TBSV

For the extraction of TBSV, 200 µg of infected plant material was used, while the purification was done using 1 g of material. The extraction and purification of viral particles were adapted from previous literature (Grasso et al., 2013). Briefly, plant material was ground and homogenized with an extraction buffer consisting of 50 mM Na-acetate pH 5.3, 1% ascorbic acid, and protease inhibitors. Plant material was homogenized with 3 mL/g in case of VNPs purification and 2 mL/g in case of VNPs extraction for quantification in agarose gels (Section 2.4). After incubation on ice for 1 hour, followed by filtration using filters with a porosity of 0.8 µm, the extract was centrifuged at 8,000 g for 15 minutes at 4°C. The supernatant was used for the quantification of VNPs in agarose gels. For further VNPs purification, the supernatant was additionally centrifuged at 90,000 g for 1 hour at 4 ˚C to finally precipitate the viral particles. The pellet was resuspended in 0.7% NaCl and centrifuged for the last time at 8,000 g for 15 minutes at 4°C. Protein content in the supernatant was quantified by Bradford method (Bradford, 1976) and used for the infection of plants with purified TBSV viral particles (VNPs).

### 2.4 Gel electrophoresis and quantification of viral particles

The method used for detecting and visualizing VNPs was adopted from recent literature (Pivotto et al., 2025). Briefly, viral particles were extracted from infected leaf material as described in Section 2.3 and then mixed with loading buffer (50 mM Tris-HCl pH 6.8, 10% glycerol, 18 mg bromophenol blue, 6X stock) added before protein electrophoresis in a 1% agarose-TAE buffered gel to quantify VNPs. As a reference, a known amount of purified TBSV.pLip particles was used. Electrophoresis was conducted in a TAE buffer at 120 V for 1 hour. Overnight staining with Quick Coomassie Stain (Neo-Biotech, France) was used for the visualization of protein bands, and their quantification was done by densitometric analysis using ImageLab software (Bio-Rad, USA).

Quantification of VNPs in viral sap was done similarly to the procedure described above. Infected plant material was homogenized directly in PBS instead of the extraction buffer and loaded with the same loading buffer previously mentioned. After the quantification, different amounts of VNPs were used to infect *N. benthamiana*. The quantities of VNPs tested were 31, 62.5, 125, 250, and 500 µg per plant. A total of six plants per condition were infected *via* spray. As a positive control, two *N. benthamiana* plants for each amount were infected *via* method described in section 2.2.

### 2.5 Statistics

The statistical significance of the amount of accumulated VNPs in sampled plant material (sample consists of 6 plants ground together, and the positive control includes 2 plants ground together, both with 3 replicates) was evaluated by comparing the results obtained in the same experiment, running Tukey-Kramer multiple comparison tests. Statistically significant variations with a p-value <0.05 are marked with different letters.

## 3 Results

### 3.1 Optimization of infection method

*N. benthamiana* is a well-known system used for approaches of plant molecular farming, and it can be inoculated with viral vectors by using spraying methods (Monroy-Borrego and Steinmetz, 2022). Recently, an engineered TBSV virus was used to infect *N. benthamiana* for the production of VNPs displaying the immunodominant peptide Lip1 (Zampieri et al., 2020). It is worth considering that plants showed signs of infection on apical leaves 4 days after TBSV inoculation, while after 10 to 14 days they were completely necrotic (Dildabek et al., 2021).

**Figure 1:**
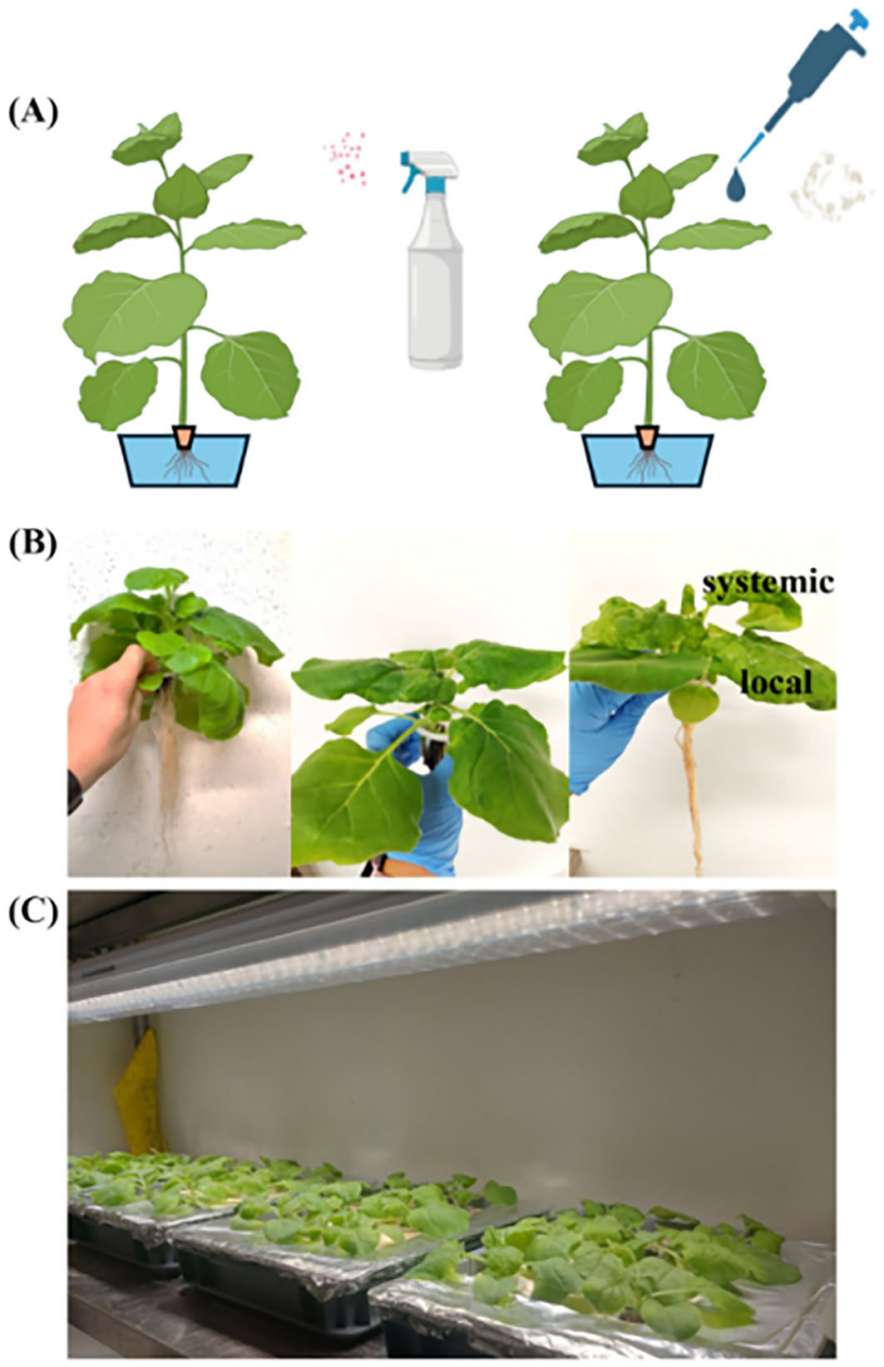
Spraying and conventional method using celite (diatomaceous earth) for the infection of *N. benthamiana*, respectively. Picture created with https://Biorender.com**(A)**. Phenotypic traits of infection with TBSV virus in hydroponic *N. benthamiana* with representation of local and systemic infection **(B)**. Specifically, from left to right: not infected *N. benthamiana*, infected via spray with TBSV WT, positive control plant infected with TBSV WT. Picture of the incubation chamber for infected hydroponic plants **(C)**.

To develop a TBSV infection process of *N. benthamiana* that can be easily implemented in an indoor cultivation system, the possibility of infecting the plants with a simple sprayer was investigated. The spraying method was developed without the use of surfactants (**Figure 1A**), utilizing a viral sap derived from previously infected plants. The tested plants were infected as previously described, and untreated plants were used as controls (**Figure 1B**, left). No major phenotypic differences were observed comparing *N. benthamiana* plants infected via the spraying method (**Figure 1B**, middle) or the conventional viral infiltration method, with the latter used as a positive control (**Figure 1B**, right). **Figure 1C** shows where the infected plants were kept in the incubation phase for VNPs accumulation.

The efficiency of infection with only spraying of viral sap was assessed with different spraying solutions (**Supplementary Figure S3**), such as spraying a mix of viral sap and celite, spraying first a dilution of celite in PBS and then sap, or using viral sap for infection. The dilutions of viral sap used in this infection test were 1:10. As controls, two methods of infection were used, namely a conventional method of infection where celite was used for mechanical damage before application of viral sap, and a second one where celite and viral sap were applied on the leaf surface together as a mixed solution. The results presented in **Supplementary Figure S3** show the accumulation of 2.7 ± 0.3 mg/g FW of VNPs using the conventional procedure of infection. Anyway, VNPs were also significantly accumulated in plants sprayed with the sap solution. In more detail, spraying only viral sap without celite resulted in the accumulation of 1.4 ± 0.05 mg/g FW of VNPs, the highest among the two different spraying methods, potentially due to the blockage of the virus entrance when celite is applied but not spread evenly on the leaf surface. Furthermore, accumulation of VNPs in control plants was higher when celite was separately applied before viral sap than when it was spread as a mixture on the leaf. Thus, the method in which celite was first spread on the leaf surface, followed by the sap application, was used in the further tests as a positive control. Conversely, only viral sap was applied for the infection *via* spray.

### 3.2 TBSV infection with quantified particles

The relationship between the amount of VNPs used for the infection and their final accumulation in infected plant material was investigated (**Supplementary Figure S4**). Specifically, the infection of the plants was performed using VNPs purified from previously infected plant material. A positive correlation was observed between the VNPs used for the infection and the particles accumulated during the 7-day incubation period. Anyway, this correlation was only observed in control plants (**Supplementary Figure S4A**), which were infected using 2, 10 and 50 µg of VNPs. Conversely, VNPs-sprayed plants showed infection symptoms; however, the accumulation of viral particles was too low and under the limit of detection of the Bradford assay (**Supplementary Figure S4B**).

**Figure 2:**
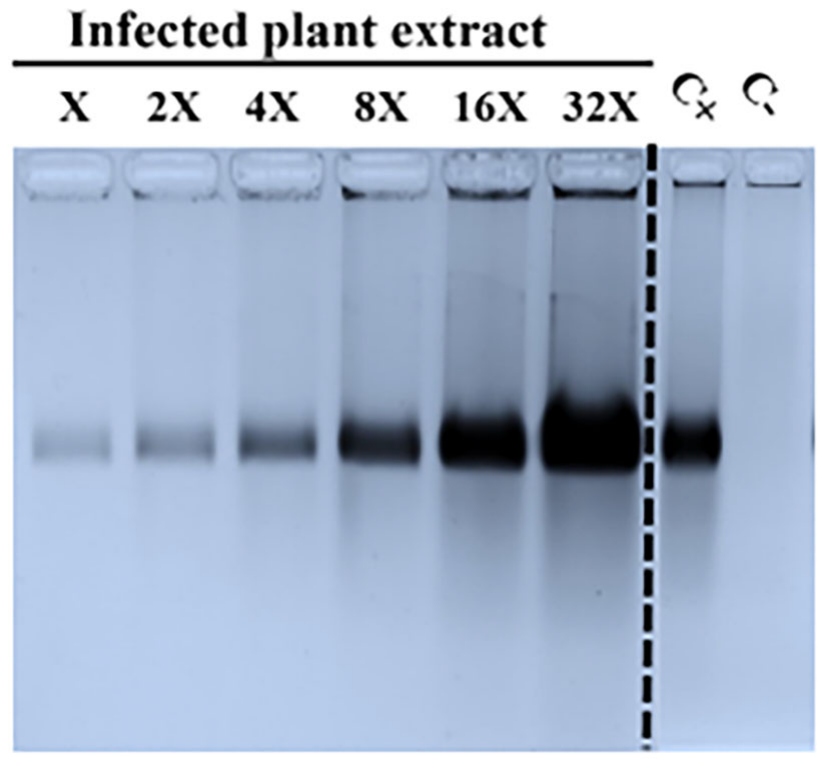
Sensitivity detection method for TBSV particles extracted from sprayed plants for TBSV infection. Extraction of VNPs was done in an acidic extraction buffer; for every 200 mg of fresh leaf material, 400 µL of extraction buffer was added. Different volumes (X = 0.47 µL) of infected plant material (TBSV WT) were loaded on the gel and stained with Coomassie staining. Infected plant material showed only one band, corresponding to the VNPs, as shown in a representative extract from infected plants (sample), while no bands were detected in the negative control.

To tackle the problem of quantification and detection of VNPs, an innovative protocol, recently described in literature (Pivotto et al., 2025), was used. **Figure 2** presents the detection capacity and sensitivity of agarose gel electrophoresis used for the separation of native VNPs extracted in acidic buffer from plants infected by spraying and then visualized with commercial Coomassie stain. Using this protocol, it was possible to detect only one band that referred to TBSV VNPs; conversely, non-infected plants (negative control) showed no bands after staining.

**Figure 3:**
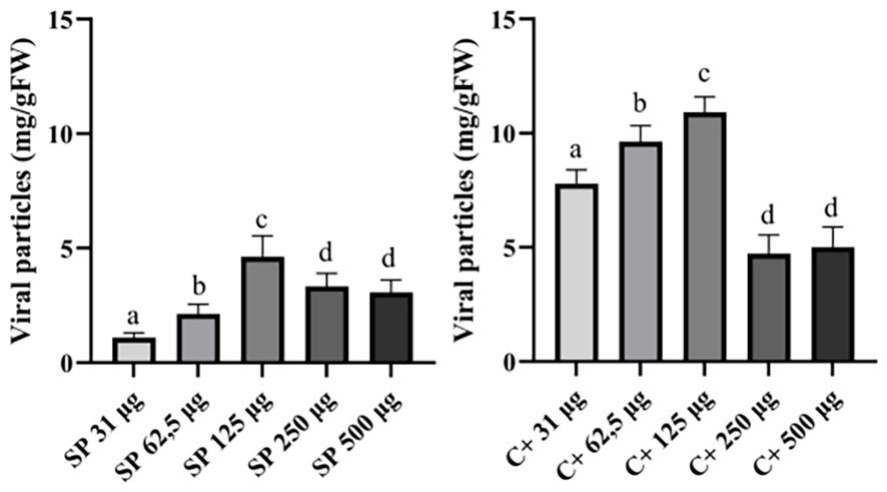
Accumulation of VNPs (mg/gFW) in *N. benthamiana* plants infected with viral sap containing a known amount of TBSV WT. The leaf material was collected after 7-dpi. Amounts of viral particles used for the infection per plant are: 31, 62.5, 125, 250, and 500 µg. Infection methods: *via* spray (left) or celite (control, right), as described in M&M. Data shown are based on quantification of the bands observed in Coomassie-stained agarose gel and analyzed by densitometric analysis. Error bars are reported as standard deviation (n = 3, mean ± SD, statistical significance is expressed with different letters according to Tukey-Kramer test, p <0.05).

To investigate the correlation between the quantity of VNPs used for infection and the amount accumulated in infected plant material, an infection test was conducted using sap containing a known amount of TBSV WT. **Figure 3** and **Supplementary Figure S5** indicate that the accumulation of VNPs in plants after 7-dpi was positively correlated with the amounts of particles used for infection in the samples containing 31, 62.5, and 125 µg of VNPs. Surprisingly, when the two highest quantities of VNPs were used for infection (250 and 500 µg), lower amounts of VNPs were detected after 7-dpi. Specifically, the excessive use of particles might have caused the lower accumulation of VNPs in the infected plants. This was likely due to the viral infection mechanisms, in which di-RNAs (defective interfering RNAs) can alter the replication of the virus and consequently its accumulation in the host cell (Havelda et al., 2005; Pathak and Nagy, 2009). Additionally, it was also shown that an excessive amount of infectious agent decreased the expression efficiency of the protein of interest in the plant host (Jin et al., 2015).

### 3.3 *N. benthamiana* infection with TBSV WT and TBSV.pLip viral sap

**Figure 4:**
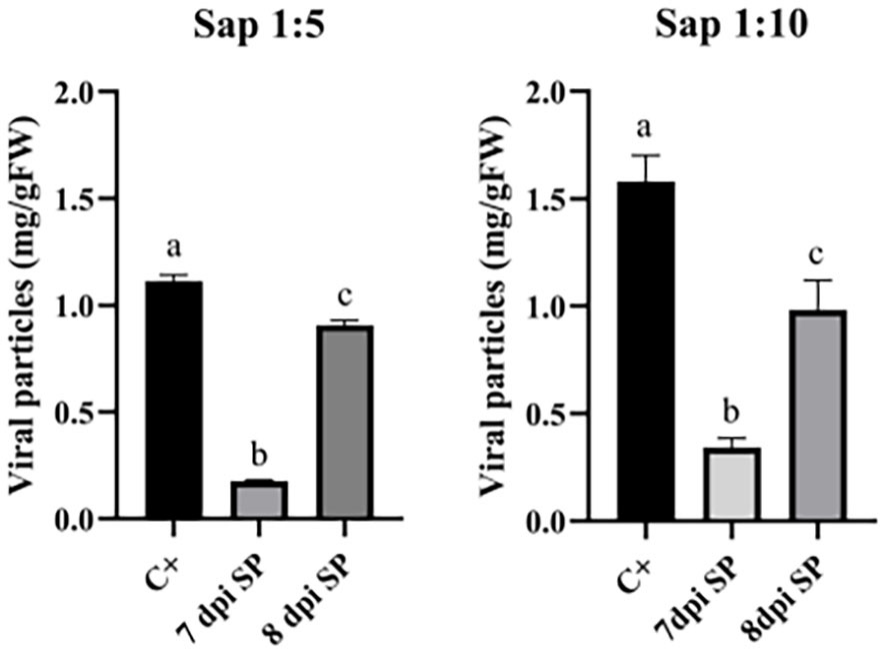
Accumulation of VNPs (mg/gFW) in *N. benthamiana* plants infected with TBSV WT viral sap in dilutions of 1:5 and 1:10 with PBS buffer. The leaf material was collected after 7 days for the C+ sample, whereas 7- and 8-dpi were evaluated for SP (sprayed) samples. Data shown are based on quantification of the bands observed in Coomassie-stained agarose gel and analyzed by densitometric analysis. Error bars are reported as standard deviation (n = 3, mean ± SD, statistical significance is expressed with different letters according to Tukey-Kramer test, p <0.05).

The infection tests with purified particles, where positive correlation was observed only in control plants (**Supplementary Figure S4A**), steered the experimental approach to avoid the costly and time-consuming purification of VNPs, instead using ground and homogenized material with PBS buffer, without VNPs enrichment. Moreover, the evidence of higher accumulation of VNPs in plants treated with the conventional procedure compared to the sprayed plants (**Figure 3**) suggested that the infection in the latter might be delayed due to a late entry of the virus into the plant inner sections. Thus, different incubation times were evaluated to increase the accumulation of particles in sprayed plants. To this end, TBSV WT and TBSV.pLip viral sap were utilized, with the latter being a promising therapeutic technology for rheumatoid arthritis (Zampieri et al., 2020). The saps in PBS buffer were used at dilution of 1:5 and 1:10 (ratio g fresh biomass/mL solvent) and sprayed on *N. benthamiana* plants. Leaf material was collected after a 7- or 8-dpi, and the accumulation of viral particles was evaluated. It is worth mentioning that the collected leaf samples were locally and systemically infected due to the possibility of TBSV to move through the plant tissue (Qu and Morris, 2002). As a proof of concept, **Figure 4** shows the infection with TBSV WT viral sap in 1:5 and 1:10 PBS buffer and the accumulation of VNPs after 7- and 8-dpi. Significant differences were observed between the accumulation in samples collected on days 7 and 8 (**Supplementary Figure S6**), meaning that the accumulation of VNPs can be increased with longer incubation time. Control plants still show the highest accumulation among all treatments.

As TBSV WT was used as proof of concept for the development of spraying and quantification methods, the same experiment was also conducted using the TBSV.pLip construct expressing the peptide of interest. Moreover, incubations longer than 8-dpi were also evaluated to reach high accumulation in sprayed samples.

**Figure 5:**
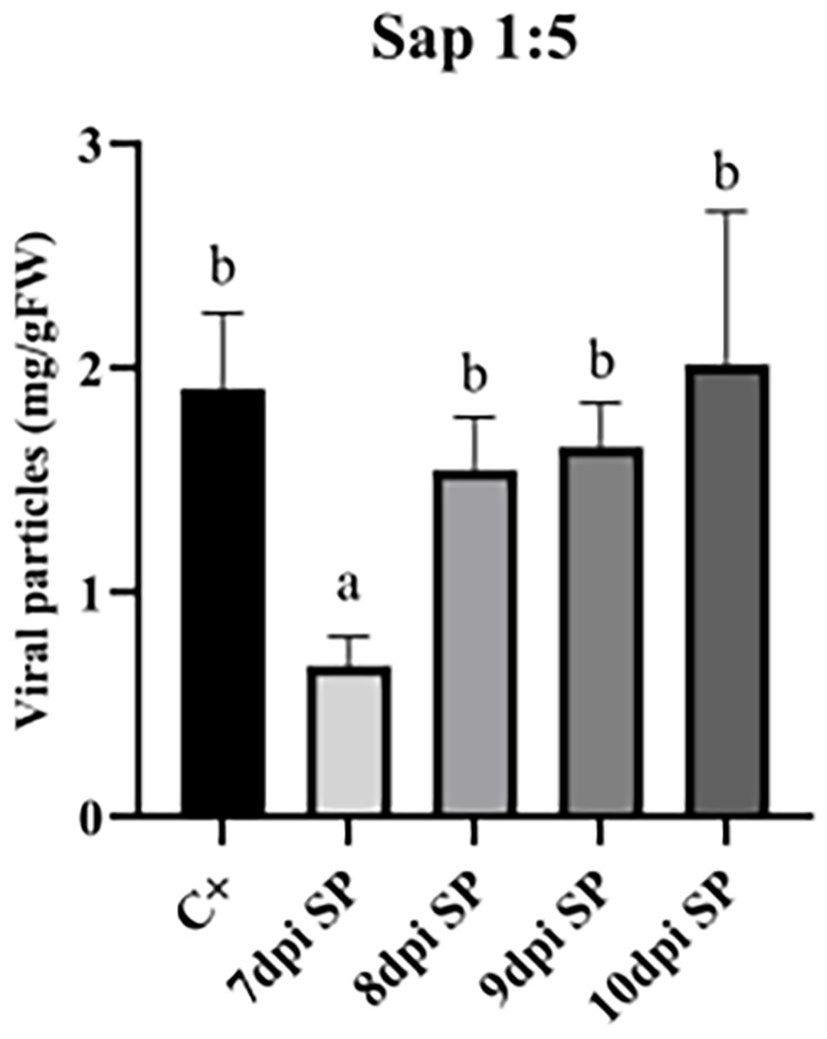
Accumulation of VNPs (mg/gFW) in *N. benthamiana* plants infected with TBSV.pLip viral sap in dilution of 1:5 with PBS. The leaf material was collected after 7-, 8-, 9- and 10-dpi. Data shown are based on quantification of the bands observed in Coomassie-stained agarose gel and analyzed by densitometric analysis. Error bars are reported as standard deviation (n = 3, mean ± SD), and the statistical significance is expressed with different letters according to Tukey-Kramer test (p <0.05).

To this aim, *N. benthamiana* plants were sprayed with a 1:5 dilution of TBSV.pLip-viral sap in PBS and collected after 7-, 8-, 9- and 10-dpi (**Figure 5**). It was observed that sprayed samples collected at 8-dpi or longer had comparable accumulation of VNPs to samples infected with the conventional approach (**Supplementary Figure S7**), where the entrance of the virus in plant tissue was facilitated with celite (p<0.05). Sprayed plants at 9-dpi accumulated 1.6 ± 0.19 mg/gFW of VNPs, while control plants reached 1.9 ± 0.33 mg/gFW at 7-dpi. The accumulation of VNPs in plants at 10-dpi showed a large variability, possibly due to the occurring necrosis in the leaf biomass.

As evidenced in **Figure 5**, the higher accumulation of particles at 8-dpi was confirmed. Moreover, it was observed that the best accumulation of VNPs occurred with post-infection incubation time between 8 and 9 days, resulting in ~1.55 and 1.65 mg/gFW, respectively. **Supplementary Figure S8** shows that there are no major changes in phenotype between the plants at 8, 9, and 10 dpi. This agrees with the lack of statistical difference in the accumulation of VNPs in plants collected at these time points. Control plants are always collected after 7-dpi, as a longer period was not possible due to leaf necrosis. Although it has been reported that necrosis in plants can be postponed with anti-necrotic substances (Norkunas et al., 2018), such as lipoic acid, ascorbic acid, and polyvinylpyrrolidone (PVP), these substances were not used in this work due to the efforts to keep the infection process as simple as possible and lower the costs of an already costly system.

In this work, the growth of *N. benthamiana* was tested at a lab-scale using a hydroponic system at a density of 400 plants/m^2^, with controlled environmental conditions such as light intensity, temperature, and humidity (**Supplementary Figure S1**). A single plant weighed ~10g, resulting in the growth of up to ~4 kg/m^2^. Considering that a) out of the whole plant only local and systemic leaves were collected (**Figure 1**), with the biomass of ~1.3 g/plant (systemic + local), b) *N. benthamiana* took 4 weeks to grow from seeds to the plant of desired size in the tested conditions, and c) a year had 49 operational weeks, and 3 weeks dedicated to maintenance and possible breaks in production, it was possible to hypothesize 12 cycles of cultivation per year, including the infection stage. Thus, the leaf biomass yearly production yield reached around ~6.25 kg/m^2^. Considering the accumulation of VNPs per fresh weight (**Figure 5**), such yearly biomass production leads to a potential VNPs yearly production of ~12.5 g/m^2^.

## 4 Discussion

Efforts presented in this work had a final goal to be implemented on a larger scale. The control method for the TBSV infection, based on the use of celite, showed the best result in terms of VNPs accumulation compared to other treatments (**Figure 3**). However, this traditional infection system is manageable only at lab-scale conditions and not easily implementable in an industrial setting. Thus, any pretreatment of plants or the use of a conventional method of infection will be excluded to obtain the simplicity needed for industrial applications.

Several successful studies have been conducted in the field of molecular farming for the production of plant-made therapeutics (Diamos et al., 2020; Rybicki, 2020; Chung et al., 2022). Notably, agroinfiltration is the most used method for further production of recombinant molecules, as it is a well-established technique for the transient infection of plants (Leuzinger et al., 2013; Norkunas et al., 2018).

Molecular farming systems that rely on the use of viruses instead of *A. tumefaciens* simplify operational processes, as there is no need to maintain bacterial culture cultivation as previously described (Monroy-Borrego and Steinmetz, 2022). Specifically, viral sap can be obtained directly from infected leaf material, and the spraying method also contributes to the system’s straightforwardness. Another advantage of using the virus compared to *Agrobacterium*-based inoculation methods is the absence of LPS (lipopolysaccharide). The latter must be removed from the final product for many applications, particularly in human therapies (Monroy-Borrego and Steinmetz, 2022). Even though *A. tumefaciens* can be sprayed on plants too, it requires adding surfactants such as Silwet-77 (Hahn et al., 2015; Jibrin et al., 2021) to facilitate the entrance of bacteria into the plant cells. Instead, as shown in this work, such surfactants are not necessary to produce the molecule of interest using TBSV. Although further research is needed, especially in the relationship between the amount of viral material used for the infection and accumulation of VNPs post-infection, this approach can be implemented in the scale-up production of pharmaceutical molecules. It has been demonstrated that the use of high-pressure spraying systems in combination with various mechanical aids, such as celite or beads, improved the transmission of plant viruses in plant material (Laidlaw, 1986; Fahim et al., 2012). However, this technology requires higher investments in terms of costs and technology compared to a fine mist sprayer, which can be easily implemented in an automated indoor system.

**Figure 6:**
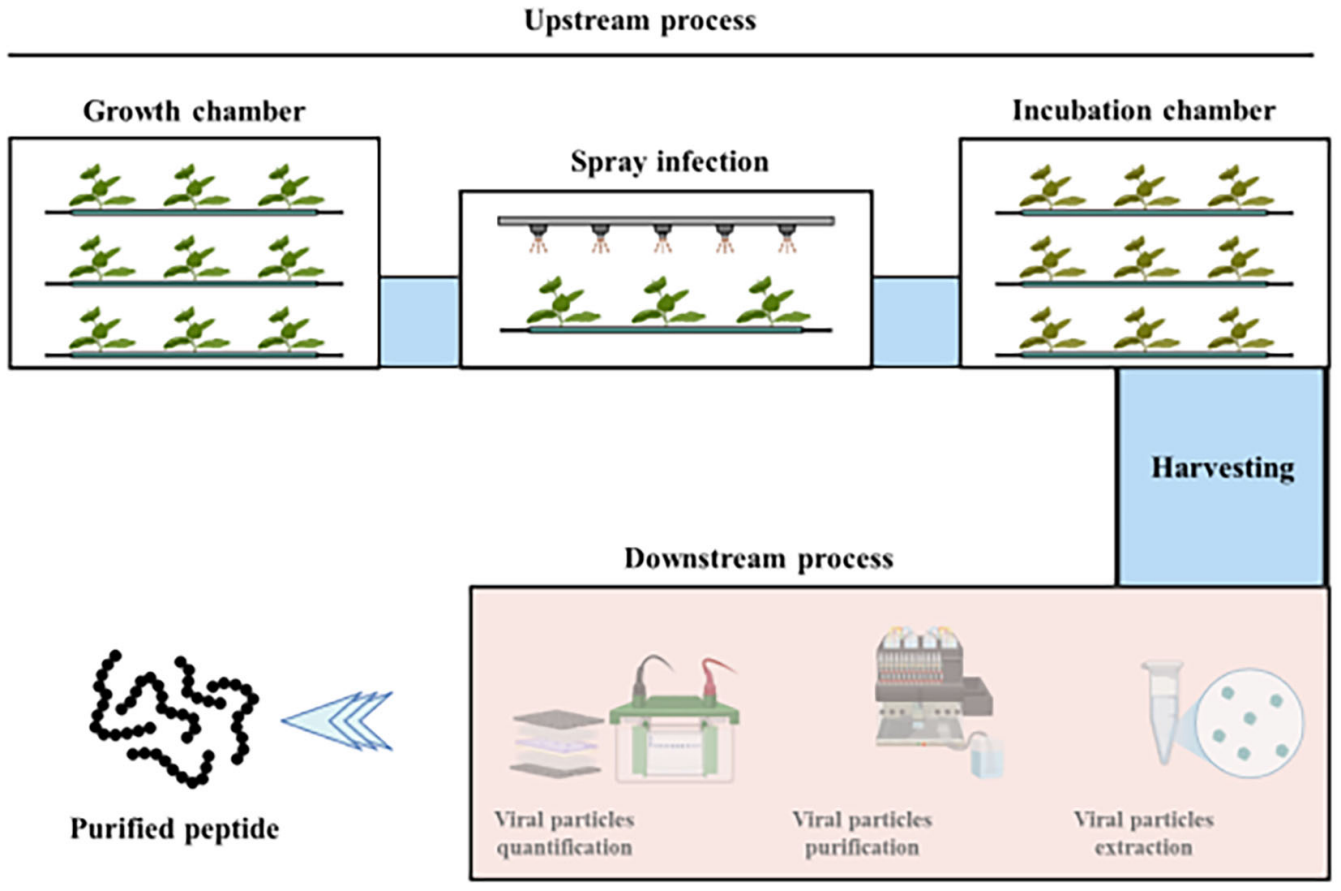
Schematic representation of an indoor system for the hydroponic cultivation of *N. benthamiana*. The plants are then infected with viral vectors for the production of recombinant proteins. Plants are first grown in a specific growth chamber until reaching the desired size. At this point, the mature plants are moved into a tunnel where they are sprayed with the viral sap. Then, the protein constructs of interest accumulate in the plant biomass kept in a specific incubation chamber. Finally, the desired protein constructs are purified from the plant material and quantified. The scheme was done using BioRender.

As mentioned in the Results section, systemically and locally infected leaves were collected, mimicking the harvesting that could be performed in an automated system such as vertical farming modules. In such modules, all the processes, from sowing seeds through infection to the collection of leaf material, are executed by automated machines (Holtz et al., 2015; Buyel, 2019). **Figure 6** describes a scheme of an indoor system used for plant molecular farming. After cultivation in the growth chamber, the plants are transferred to an infection chamber where infection is done by spraying the TBSV virus. This section must be separated from the growth room to prevent viral contamination of the growing non-infected plants. Furthermore, the infection chamber can be separated from the incubation chamber. In particular, the infection chamber is a tunnel with a conveyor track, where trays with plants are sprayed with the virus. Then plants are kept in the incubation chamber for VNP accumulation. The harvesting of locally and systemically infected leaves can be done after 7–9 days, depending on the VNP accumulation and the health state of plants. Next steps include VNP extraction/purification and quantification to obtain the peptide of interest. A significant part of the process being automated can help reduce production costs, which are already very high for indoor systems, as reported in literature (Tusé et al., 2014; McNulty et al., 2020).

Considering that the cultivation system showed a fresh leaf biomass productivity of ~6.25 kg/m^2^/year at lab scale, and that ~2 mg/gFW of VNPs were produced in the mentioned lab-scale settings (**Figure 5**, 9-dpi), it is possible to estimate a productivity ~1 g of VNPs per m^2^ and ~12.5 g of VNPs per m^2^/year. From such 12.5 g of VNPs, it could be possible to extract ~0.4 g/m^2^/year of highly-pure Lip1, taking into account that 50µg of VNPs have 4*10^12^ individual particles and 1.6 µg of Lip1 peptide. Monroy-Borrego reported a yield of 0.3-0.5 mg/gFW of TMV (Tobacco mosaic virus) particles when *N. benthamiana* plants were infected with the spraying method, while slightly lower yield (0.17-0.28 mg/gFW) was obtained when TMV-Lys mutant was used for spray infection of plants (Monroy-Borrego and Steinmetz, 2022).

Additionally, to ensure that the process produces the highest quality VNPs, the entire production process must comply with current Good Manufacturing Practices (cGMP), even though the upstream process is allowed to be non-GMP (Schillberg and Finnern, 2021). Overall, the upstream and downstream processes require additional testing and monitoring systems.

In conclusion, the presented work aimed to simplify the production process of VNPs derived from TBSV host in *Nicotiana benthamiana*. The conventional infiltration method is time-consuming and requires qualified personnel to perform the infection, whereas the method developed by spraying viral sap can be easily and simultaneously performed by automated machines on a large number of plants. Automation of the process can reduce the number of workers needed to perform infiltration, and it further decreases operational costs, as sprayers are cost-effective devices and usually already present in indoor cultivation systems. Overall, the results obtained in this work showed that infection by spray initiated later compared to the conventional method due to the delayed entrance of the virus; on the other hand, it can reach an accumulation of VNPs similar to that obtained in control plants, if the cycle is prolonged by just one or two days. The lower time efficiency presented here can compensate for the complexity and time consuming of the conventional process. Moreover, it can be easily scaled to increase the production yield of the system, especially when performed in a controlled environment, such as a vertical farming system.

## Data Availability

The original contributions presented in the study are included in the article/**Supplementary Material**. Further inquiries can be directed to the corresponding authors.
