# Supplementary material for: An innovative infection method for the accumulation of viral nanoparticles in Nicotiana benthamiana
Source: Front Plant Sci. 2025 Nov 26;16:1727190. doi: 10.3389/fpls.2025.1727190 (PMC12689547; doi:10.3389/fpls.2025.1727190)
Supplement: Supplementary file 1 [file DataSheet1.docx]

Supplementary Material

An innovative infection method for the accumulation of viral nanoparticles in *Nicotiana benthamiana*.

Kristina Ljumović, Anthony Rosa, Alessia Raneri, Matteo Ballottari, Linda Avesani*, Nico Betterle*

*** Correspondence:** linda.avesani@univr.it; nico.betterle@univr.it


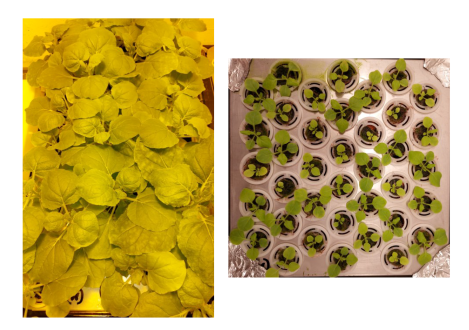


**Supplementary Figure 1.** *Nicotiana benthamiana* hydroponic cultivation. Estimated plant density of 400 plants/m^2^. Cultivation of plants, from seedlings to the infection step, was done in metal grids provided by ONO Exponential Farming (Italy)


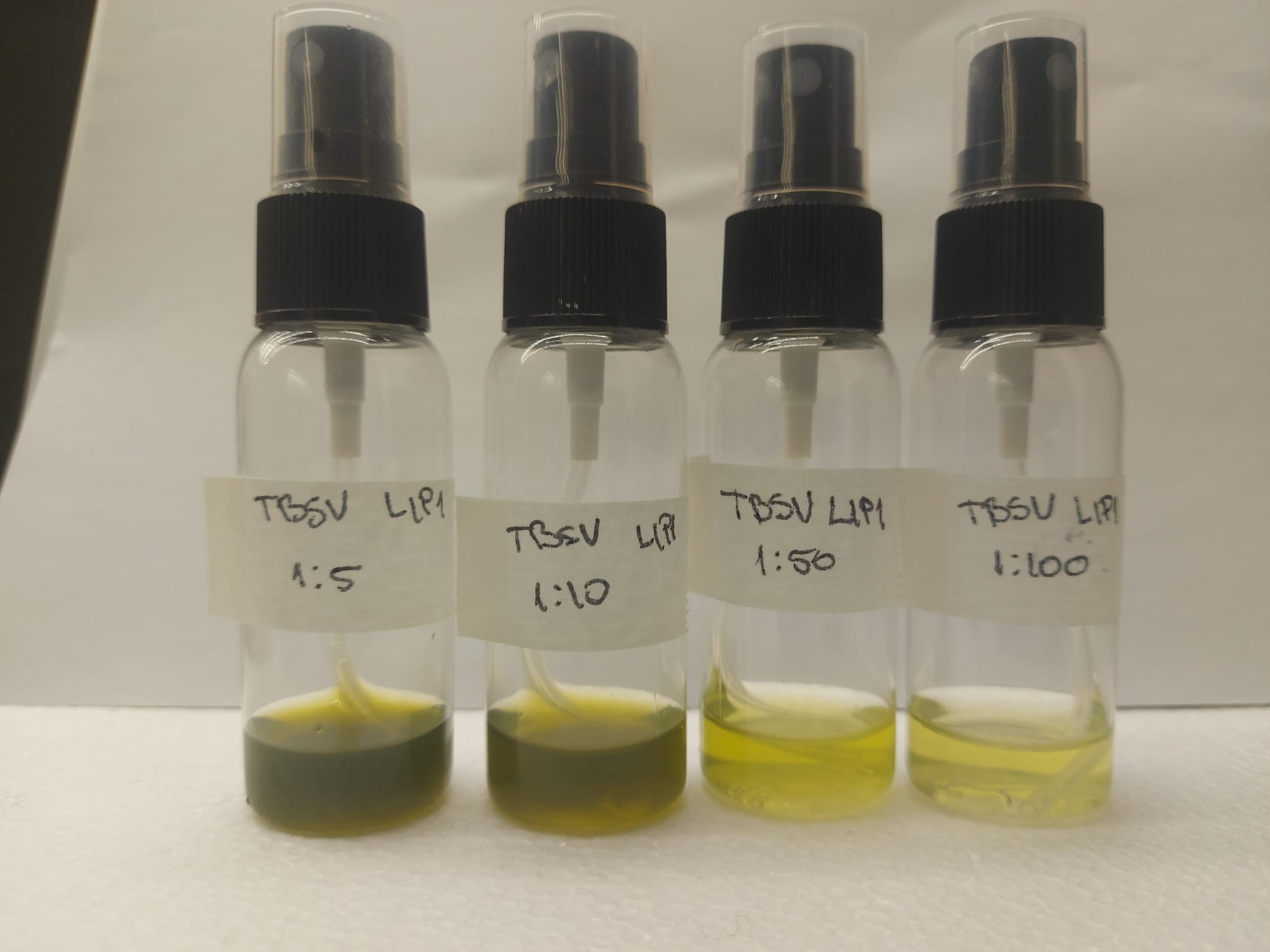


**Supplementary Figure 2.** Fine mist sprayer used to inoculate *N. benthamiana* with sap containing TBSV. Saps were obtained as described in section 2.2.


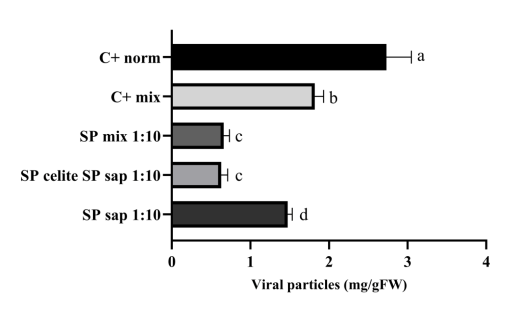


**Supplementary Figure 3.** Different methods for *N. benthamiana* infection with TBSV sap dilution 1:10. *C+ norm*, plants infected as positive control with the method described in M&M; *C+ mix*, plants infected with the mixed solution of celite and viral sap; *SP mix 1:10*, plants sprayed with the mixed solution of celite and sap; *SP celite SP sap mix 1:10*, plants sprayed with solution of celite and then sprayed with viral sap; SP sap 1:10, plants sprayed only with viral sap as described in M&M. Accumulation of viral particles is presented as mg/gFW of leaves (FW, fresh weight). Data shown are based on quantification of the bands observed in Coomassie-stained agarose gel and analyzed by densitometric analysis. Error bars are reported as standard deviation (n = 3, mean ±SD, statistical significance is expressed with different letters according to Tukey-Kramer test, p <0.05).


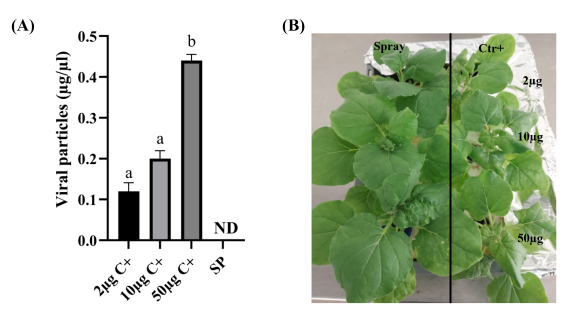


**Supplementary Figure 4.** Accumulation of VNPs in *N. benthamiana* control plants infected with purified VNPs (2 µg, 10 µg, and 50 µg). The concentration of purified VNPs was expressed in µg/µL (**A**). The infection method is described in M&M. A positive correlation between the amount of particles used for the infection (X axis), and the amount of particles accumulated (Y axis) is evidenced. Data shown are based on quantification of purified VNPs using Bradford method. Error bars are reported as standard deviation (n = 3, mean ±SD, statistical significance is expressed with different letters according to Tukey-Kramer test, p <0.05). Spraying with purified particles showed symptoms of infection, but VNPs accumulation was not detectable (ND) (**B**).


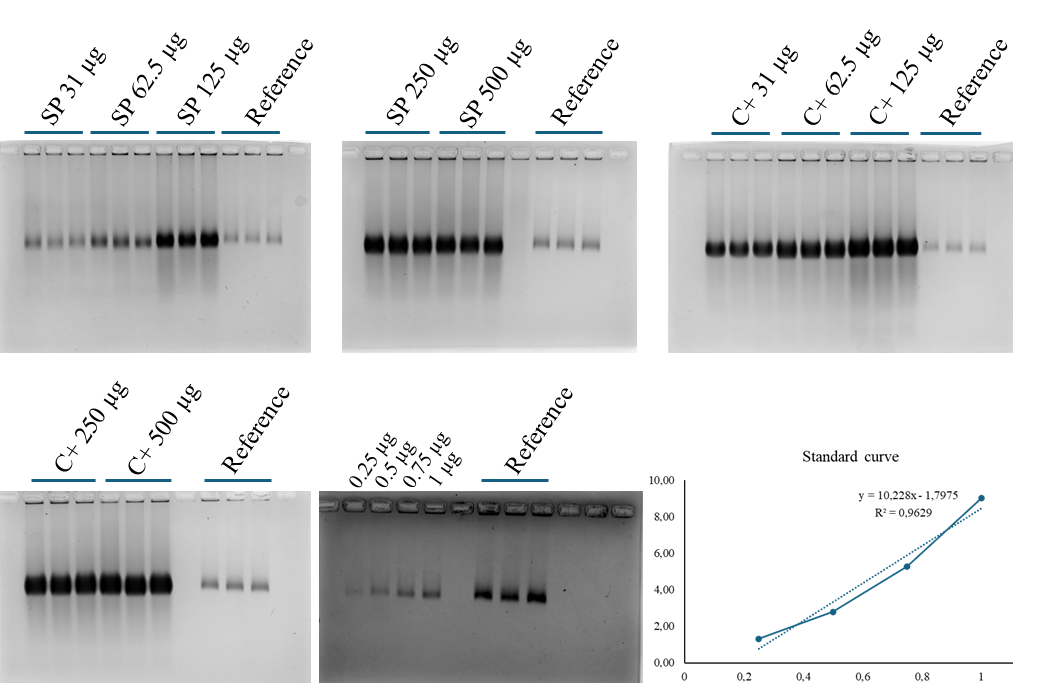


**Supplementary Figure 5**. Coomassie-stained agarose gels and standard curve used for the quantification by densitometric analysis of data shown in Figure 3. Standard curve was made loading known amounts (0.25 µg, 0.5 µg, 0.75 µg, 1 µg) of purified TBSV.pLip (R^2^=0.9629). As a Reference, samples containing 2.3 µg of purified TBSV.pLip were loaded in every gel for cross-quantification of samples distributed in different gels. 5µl of C+ and 7.5µl of SP sample extracts were loaded in agarose gels.


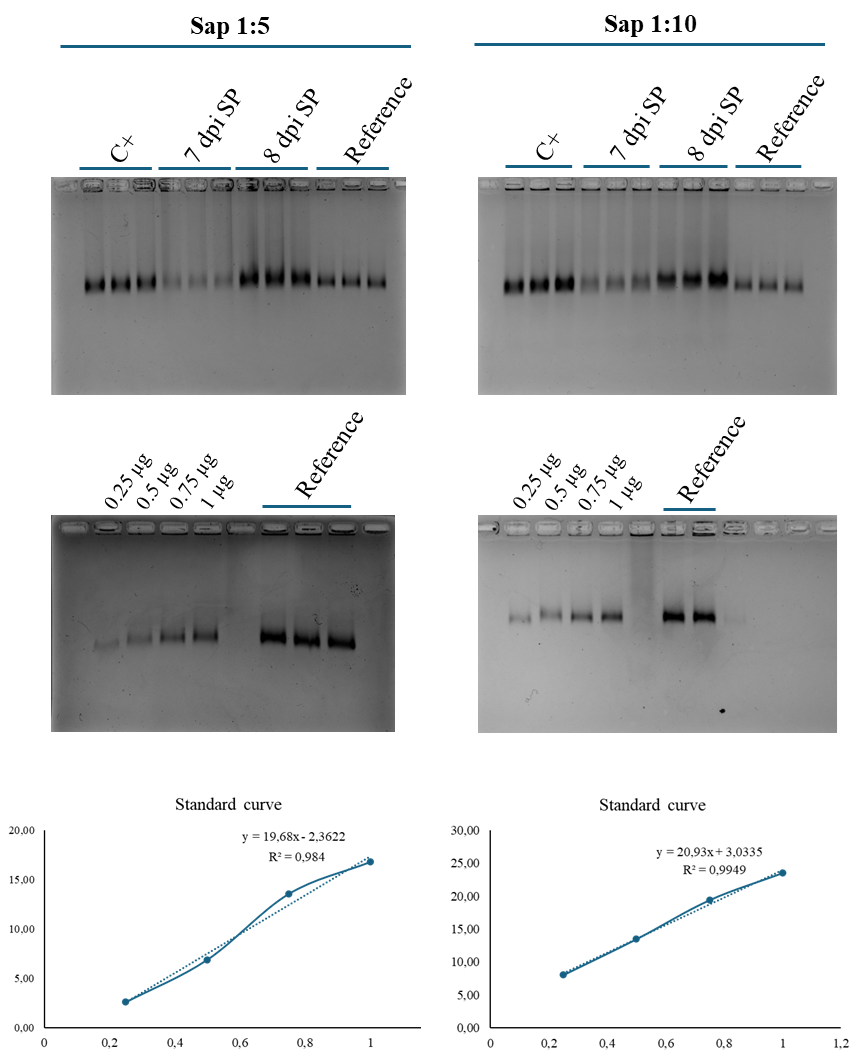


**Supplementary Figure 6**. Coomassie-stained agarose gels and standard curves used for the quantification by densitometric analysis of data shown in Figure 4. Standard curves were made loading known amounts (0.25 µg, 0.5 µg, 0.75 µg, 1 µg) of purified TBSV.pLip (R^2^=0.984 in “Sap 1:5” and R^2^=0.9949 in “Sap 1:10”). As a Reference, samples containing 1.6 µg of purified TBSV.pLip were loaded in every gel for cross-quantifications of samples. 5µl of C+ and 7.5µl of SP sample extracts were loaded in agarose gels.


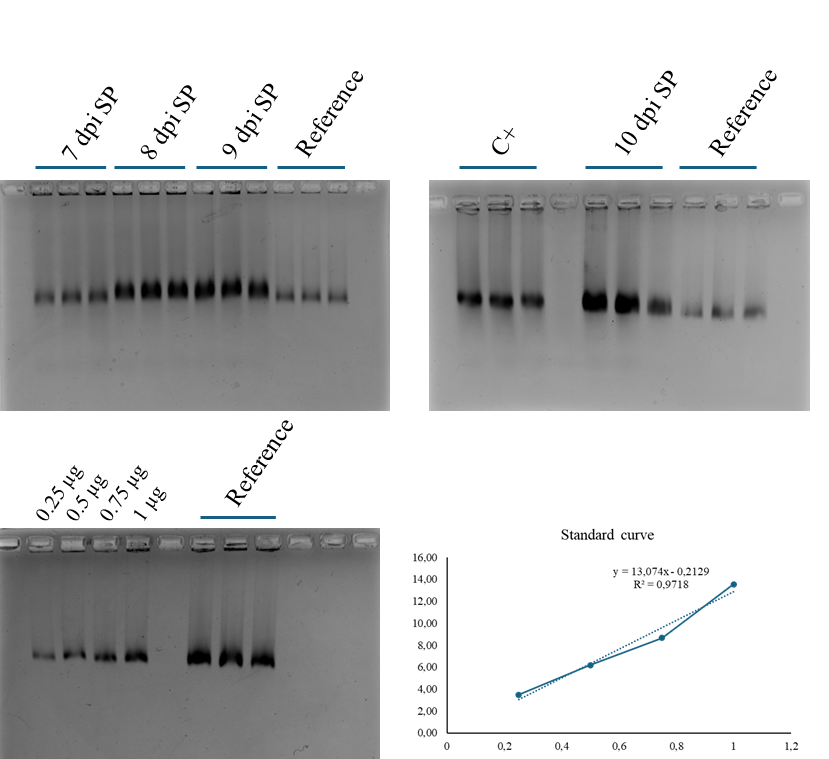


**Supplementary Figure 7**. Coomassie-stained agarose gels and standard curve used for the quantification by densitometric analysis of data shown in Figure 5. Standard curve was made loading known amounts (0.25 µg, 0.5 µg, 0.75 µg, 1 µg) of purified TBSV.pLip (R^2^=0.9718). As a Reference, samples containing 1.6 µg of purified TBSV.pLip were loaded in every gel for cross-quantifications of samples distributed in different gels. 5µl of C+ and 7.5µl of SP sample extract were loaded on agarose gels.


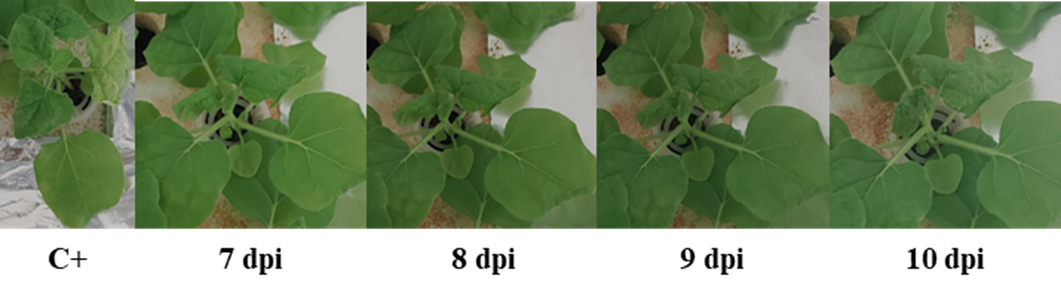


**Supplementary Figure 8.** Phenotypic traits of a representative plant infected with sprayed TBSV.pLip sap from 7 to 10 dpi, including the plant (C+) that was infected *via* method described in M&M for control plants, at 7dpi.
